# Language proficiency and academic achievement in rural and agricultural Latine youth: A mixed methods approach

**DOI:** 10.1111/jora.70166

**Published:** 2026-03-23

**Authors:** Alejandro Baquero‐Sierra, Zoe E. Taylor, Alexia Carrizales, Yumary Ruiz

**Affiliations:** ^1^ Department of Curriculum and Instruction Purdue University West Lafayette Indiana USA; ^2^ Department of Human Development and Family Science Purdue University West Lafayette Indiana USA; ^3^ Department of Public Health Purdue University West Lafayette Indiana USA

**Keywords:** academic success, behavioral and emotional problems, cognitive executive functions, English language learners, Latine adolescents

## Abstract

This study investigates the influence of executive functioning, language proficiency, and behavioral–emotional challenges on the academic performance of rural immigrant Latine youth in the Midwest. Using a convergent mixed methods design, we integrated quantitative analyses (*N* = 178) of academic indicators (GPA, Math, and ELA scores) with qualitative interviews (*n* = 47) that examined students' lived experiences. Higher behavioral difficulties were associated with lower academic outcomes, while executive functioning predicted academic success. Qualitative findings revealed that behavioral challenges often stemmed from adaptive responses to language barriers, academic stress and social exclusion. These patterns suggest that the cognitive load of second‐language acquisition influences student engagement in ways not fully captured by standardized assessments. Results highlight the importance of culturally responsive interventions that support bilingual instruction, executive functioning, and mental health to promote resilience among Latine ELL students.

English Language Learners (ELLs) in the United States are a diverse and growing student population with limited English proficiency and varied linguistic backgrounds (Bialik & Fry, 2018). They comprise 10.6% of the student population, primarily in urban (9.8%), suburban (7.9%), and smaller rural areas (4.8%, National Center for Education Statistics, 2021, Pew Research Center, 2023). Most ELL students are of Hispanic origin, and 76.4% speak Spanish as their native language. Their academic outcomes vary considerably due to multiple factors influencing school performance, particularly in middle and high school. Language barriers restrict their ability to understand instructions, participate in class, and master complex concepts (Gándara & Contreras, 2009). Cultural differences and adjustment to unfamiliar educational systems can also be overwhelming (Olsen, 2014), especially when teaching styles and classroom dynamics differ from prior experiences (Abedi, 2009). Limited background knowledge of U.S. history or social contexts further contributes to academic gaps (August & Shanahan, 2017; Callahan, 2013). In addition, restricted access to early childhood education or limited formal schooling in students' countries of origin may hinder progress (Espinosa, 2013).

ELL students in rural and new immigrant communities often face additional structural barriers, such as limited bilingual education, youth programs, and healthcare access, which affect school readiness and well‐being. These risks intersect with cognitive, behavioral, and emotional domains critical to academic development. Among Latine ELL populations, children from migrant farmworker families encounter particularly high adversity, including frequent school disruptions, poverty, and social marginalization (Free et al., 2014; Lundy‐Ponce, 2010; Taylor et al., 2024; Taylor & Ruiz, 2017; Taylor et al., 2019). Approximately 84% of students enrolled in the Migrant Education Program (MEP) are classified as ELLs (U.S. Department of Education, Office of Migrant Education, 2023). These students often experience limited access to quality instruction, high mobility, and socioemotional stress, resulting in persistent gaps in math and reading (Free et al., 2014; Taylor & Ruiz, 2017). These challenges are compounded by broader socioeconomic constraints, including low income, limited parental education, and unstable agricultural work (Crockett et al., 2016).

Rural new‐immigrant destinations heighten these risks due to geographic isolation, institutional underinvestment, and restricted access to bilingual and mental health services (Stein et al., 2016; Raffaelli et al., 2016). These conditions intensify academic disengagement and emotional strain while increasing the cognitive demands of second‐language acquisition (Suárez‐Orozco et al., 2018). At the same time, rural Latine families demonstrate important protective assets, such as intergenerational bonds and cultural resilience, which can buffer stress (Crockett et al., 2016).

Understanding these risk and protective factors is essential for contextualizing Latine students' developmental trajectories in nonurban regions, where opportunity structures differ from urban settings. However, research continues to focus largely on urban and Southwestern populations, limiting generalizability to students in rural Midwestern and Southeastern communities. Expanding this evidence base requires attention to the unique convergence of linguistic, socioemotional, and structural factors shaping academic performance in rural contexts.

## THE IMPORTANCE OF COGNITIVE AND EXECUTIVE FUNCTIONS FOR ACADEMIC SUCCESS

Cognitive and Executive Functions (CEF), including vocabulary, working memory, cognitive flexibility, and inhibitory control, are central to academic success because they support self‐regulation and effective learning strategies. These functions enable attention control and goal‐directed problem‐solving, such as reasoning, planning, and adaptive coping (Kashdan & Rottenberg, 2010). Working memory allows students to retain and manipulate information, cognitive flexibility supports perspective‐shifting, and inhibitory control reduces distraction (Kashdan & Rottenberg, 2010). Together, these abilities help regulate thoughts, emotions, and behavior, thereby facilitating learning and achievement (Roebers, 2017).

CEF development is highly sensitive to environmental stress. Conditions such as poverty, chronic stress, limited educational opportunities, and inconsistent caregiving can undermine self‐regulation (King et al., 2013), making executive functioning both vulnerable and essential among high‐risk youth (Blair & Diamond, 2008). At the same time, bilingualism may enhance cognitive flexibility and attentional control, particularly when managing multiple language systems (Ye et al., 2016). When executive functioning is compromised, students experience difficulties sustaining attention, solving problems, and adapting to academic demands (Lawton & Gerdes, 2014). Within Latine families, economic hardship and acculturative stress influence parenting organization and support for self‐regulation, although familism and consistent guidance can buffer these risks (Crockett et al, 2016; Stein et al., 2016; Kim et al., 2024).

Research consistently links CEF, especially cognitive flexibility and inhibitory control, to academic success among Latine youth, mirroring broader developmental trends. Executive functioning predicts achievement in reading (Ahmed et al., 2021; Greenfader, 2017, 2019; Jacobson et al., 2017) and mathematics (Lemberger et al., 2018; Matthews, 2018; Magalhães et al., 2020; Bukva & Memisevic, 2024; Coulanges et al., 2021). Cognitive flexibility predicts literacy and mathematics achievement even after accounting for other skills (Magalhães et al., 2020), and inhibitory control supports math performance by facilitating complex processing (Coulanges et al., 2021; Oberle & Schonert‐Reichl, 2013). Among children of migrant farmworkers, executive functioning is related to academic efficacy and resilience (Taylor & Ruiz, 2019), although socioeconomic adversity threatens both the development of executive skills and academic achievement (Li‐Grining et al., 2022).

Recent evidence also shows that bilingualism, sociocultural context, and discrimination‐related stress shape CEF. Spanish–English bilingual youth demonstrate enhanced flexibility and inhibitory control compared to their monolingual peers, even after controlling for socioeconomic factors, suggesting that bilingualism strengthens attentional regulation (Carlson & Meltzoff, 2008; Nguyen et al., 2024). For Mexican‐origin adolescents, bilingual brokering can buffer discrimination‐related cognitive stress (Kim et al., 2024). Early vocabulary exposure and language‐rich family interactions further predict inhibitory control, linking language experience with self‐regulatory growth (Peredo et al., 2015).

## BEHAVIORAL AND EMOTIONAL PROBLEMS IN LATINE YOUTH

Behavioral and Emotional Problems (BEP) among Latine youth are often misunderstood as personal deficiencies rather than as adaptive responses to intense stressors, like immigration challenges, poverty, and linguistic barriers (Taylor et al., 2019). Adverse experiences often lead to difficulties with attention, emotional regulation, and academic engagement, and can be exacerbated in rural contexts with limited resources (Free et al., 2014). Researchers have consistently linked BEP to lower academic performance in core domains requiring sustained attention and cognitive engagement, such as math and reading (Horan et al., 2016; Wickrama et al., 2016). Importantly, recent findings emphasize that BEP can manifest as contextually driven reactions to social exclusion, school‐related bullying, and the demands of navigating a second language, rather than as permanent behavioral deficits (Carrero et al., 2017; Li‐Grining et al., 2022).

Research further indicates that systemic factors, such as strained teacher–student relationships and inadequate access to mental health resources, worsen the impact of BEP on students' academic trajectories (Shi et al., 2021; Slopen et al., 2023). Despite these challenges, Latine students often display remarkable resilience and adaptability (Suárez‐Orozco et al., 2018), a quality that is rarely recognized in deficit‐oriented frameworks. Collectively, these findings highlight that BEP in Latine youth should be interpreted within the broader context of social and economic adversity, rather than as isolated indicators of dysfunction. Adopting this strengths‐based perspective is essential to understanding how these youth navigate complex educational environments while striving for academic success.

## THE PRESENT STUDY

Given the substantial challenges that Latine ELL students encounter, particularly those from farmworker and rural backgrounds, this study emphasizes the need for a nuanced understanding of how cognitive, behavioral, and emotional factors intersect to influence academic outcomes. In this context, examining the roles of both internal and external factors in shaping the academic performance and well‐being of Latine ELL students becomes essential. Consequently, the present study aims to explore these dynamics in depth, employing a mixed‐methods approach to provide a comprehensive understanding of the academic experiences of Latine students and the systemic factors that impact their educational achievement.

We combined quantitative and qualitative approaches in a convergent mixed‐methods design utilizing multiple reporters. The quantitative component investigated associations between CEF, BEP, and demographic variables (ELL status, socioeconomic status, gender, and age) and academic performance measures (Math and English language arts test scores and GPA) in a sample of rural Latine adolescents. We hypothesized that CEF, particularly cognitive flexibility and inhibitory control, would positively predict academic achievement, whereas BEP would be negatively associated with academic performance. Qualitative data explored how rural Latine youth (ELL and non‐ELL) experience and navigate linguistic barriers, BEP, and executive functioning demands within Midwestern English‐only school environments.

## METHOD

### Participants and procedure

Data for this study were drawn from the Purdue Puentes Project, a mixed method longitudinal study of Latine youth aged 10–15 years at W1 (*N* = 307, *M* = 12.21 years, 51% boys) and their primary caregivers (*N* = 288, 88.2% mothers) from rural and agricultural families in the U.S. Midwest. Following institutional review board approval from Purdue University, families were recruited through community organizations and schools serving Latine populations, such as Migrant Education Programs (MEPs), schools, and university‐affiliated Extension offices in Indiana, with a small number recruited from MEPs in Michigan. Eligible families met the following criteria: (a) self‐identified as Latino/a, (b) had a child aged 10–15 years, and (c) either participated in an MEP, worked in agriculture, or lived in a rural location. Wave 1 (W1) data were collected from 2021 to 2023 and included surveys, biometric assessments, and executive functioning tasks (predictor variables, Time 1). Academic test scores (outcome variables) were collected annually by the State Department of Education between 2022 and 2024. Predictor variables were paired with academic scores from the following year (Time 2). In addition, a subset of youth, mainly from MEP programs (*n* = 47, 50% boys; *M* = 11.74 years, 63% U.S.‐born, 95% in MEP), participated in semistructured qualitative interviews during the summer of 2021.

Survey‐based data (90–120 min) were collected in person at MEP sites, community locations, and participants' homes by trained bilingual research assistants (RAs). Before data collection, informed consent was obtained from caregivers and assent from the youth. Participants completed surveys and cognitive tasks on iPads in either English or Spanish, according to their preference; most caregivers and some youth chose Spanish. RAs assisted as needed, including reading items aloud to ensure comprehension. Youth were invited to participate in qualitative interviews using purposive sampling to ensure variation across gender, developmental stage, and migration history. Sampling continued until data saturation (Guest et al., 2006). Bilingual or monolingual RAs conducted semistructured interviews (60 min) in private locations in their preferred language (English or Spanish). The research team shared cultural and linguistic backgrounds with participants and as scholars have varied professional expertise in Latine youth health and development. All RAs completed a structured training program for all aspects of data collection. All data were de‐identified to protect participant confidentiality. Participants were compensated for their time, with compensation increasing in the second year to account for inflation and community input.

#### Present study participants

The present study uses data collected at Wave 1 and outcome variables corresponding to academic scores (provided by Indiana's Department of Education, DOE) from the subsequent school year. Participants were included if they had full scores from the Indiana Department of Education for standardized English Language Arts (ELA) and Math exams, and a Grade Point Average (GPA). This resulted in a sample of 178 students (*M*_age_ = 12.32 years, *SD* = 1.52; 54% male, 46% female). In this subsample, 94% of primary caregivers (PCs) and 28% of the youth were first‐generation immigrants, primarily from Mexico (66% of PCs and 10% of the youth) and Guatemala (14% of PCs and 10% of the youth). Most PCs (63%) did not have a high school diploma, and families' mean household income ranged between $25,000 and $30,000. Most PCs (71%) were married or living with a partner. Most students (63%) were ELLs, and 26% of youth participated in an MEP. The sociodemographic characteristics of the qualitative participants were comparable to those of the larger sample, although the majority of the interviewed youth were enrolled in MEP programs.

### Positionality of the researchers

The first author is a native Spanish‐speaking immigrant with expertise in language education and child development in Latine populations. The second author is a non‐Latina White immigrant with expertise in human development and Latine youth and families. The third author is a multiracial scholar and native Spanish speaker with expertise in socioemotional development among diverse cultural populations. The last author is a native Spanish‐speaking immigrant with expertise in qualitative research, public health, and Latine communities.

### Quantitative measures

All scales used in the study had validated Spanish versions and had been previously used in Latine samples.

#### Academic performance (T2)

Academic performance indices were evaluated using student data provided by the DOE, which included Grade Point Average (GPA) and ILEARN standardized test scores in Math and English Language Arts (ELA). GPA was calculated by converting letter grades to a numeric scale from 0 to 4 (*M* = 2.75, *SD* = 0.95, range: 0–4). The ILEARN Math score evaluates mathematical knowledge, problem‐solving, and critical‐thinking skills across grades (*M* = 6467.31, *SD* = 95.90, range: 6205–6738). The ILEARN ELA measures students' skills in reading, language comprehension, and writing (*M* = 5480.26, *SD* = 90.13, range: 5268–5733). Each academic performance score used in the analysis was collected at the end of the school year by the DOE (Time 2) and matched to the predictor variables collected by this study in the previous year (Time 1).

#### Cognitive executive functions‐CEF (T1)

Cognitive executive functions were assessed using the Flanker Inhibitory Control and Attention Test (FICA), the Dimensional Change Card Sort Test (DCCS), and the Picture Vocabulary Test (PV) from the NIH Toolbox. Behavioral tasks are available in both Spanish and English, take 3–4 min to complete on an iPad, and are valid for individuals aged 3–85 (Weintraub et al., 2013). The FICA is an objective behavioral measure of inhibitory control and attention. The test requires the participant to focus on a given stimulus while inhibiting attention to stimuli (an arrow) flanking it (mean computed score = 7.87; *SD* = 1.11, range: 3.75–10). The DCCS is a measure of cognitive flexibility, where participants are shown pictures depicting objects that vary on two dimensions (e.g., colored shapes such as red rabbits and blue boats) and are told to sort them first by one set of rules (e.g., shape) and then by another (e.g., color). “Switch” trials are used, in which the participant must change the dimension being matched, thus requiring the cognitive flexibility to choose the correct stimulus quickly (Mean computed score = 7.20, *SD* = 1.68, range: 2.13–10). The PV measures receptive vocabulary and is part of the assessment of crystallized cognitive abilities. The PV test involves participants viewing a picture and hearing a word, then selecting the image that best matches the word they hear. The test adapts to the participant's performance level, adjusting the difficulty of items based on previous responses. (Mean score = 0.48, *SD* = 0.01, range: 0.45–0.50).

#### Behavioral and emotional problems‐BEP (T1)

BEP were reported separately by parents (PR) and teachers (TR) using two subscales from the Strengths and Difficulties Questionnaire (SDQ; Goodman, 2001) on a Likert scale from 1 (not true) to 3 (certainly true). General difficulties (GD) included 20 items such as “Restless, overactive, cannot stay still for long”; “Often fights with other youth or bullies them” (PR: *M* = 1.67, *SD* = 0.22, range: 1.16–2.48, Cronbach's α = 0.79; TR: *M* = 1.32, *SD* = 0.29, range: 1.00–2.30, Cronbach's α = 0.86). Prosocial behavior included five items such as “Considerate of other people's feelings”; “Shares readily with other youth, for example books, games, food” (PR: *M* = 2.49, *SD* = 0.41, range: 1.20–3.00, Cronbach's α = 0.62; TR: *M* = 2.48, *SD* = 0.46, range: 1–3, Cronbach's α = 0.82).

#### Covariates

Covariates included youth sex (boys = 1, girls = 0), age (10–15), ELL status (ELL = 1, non‐ELL = 0), and MEP status (MEP = 1, non‐MEP = 0).

### Qualitative measures

Interviews explored participants' perceptions of their school experiences, academic motivation, and the barriers they encountered to achieving their goals. All questions were open‐ended to encourage rich narrative responses. Participants were prompted to reflect on their educational experiences, school transitions, and the factors influencing their academic success. For example, youth were asked, “Okay, now let's talk about your school and schoolwork. Have you been to other schools, or do you always go to the same school?” These questions established context for their educational trajectories. They were also asked, “Tell me how you do in school in general,” which invited them to elaborate on their academic performance, study habits, and experiences in various classes. Additional probes included: “What do you like and dislike most about the school you are in or will be going to?” as well as “What or who helps you to do your best in school?” and “Who or what makes it hard for you to do your best in school?” These questions elicited detailed responses about the supports and barriers that participants perceived in their academic environments, contributing to a deeper understanding of the factors that shape their school engagement and performance.

### Data analytics strategy

This study employed a convergent mixed methods design, concurrently collecting and analyzing quantitative and qualitative data, with integration during the interpretation and discussion phases (Creswell & Plano Clark, 2017; Fetters et al., 2013). A complementary strategy was employed, wherein each method provided distinct yet interrelated insights: quantitative analyses identified measurable patterns, while qualitative interviews captured the contextualized experiences that underlay the patterns. Complementarity facilitated convergence, complementarity, and expansion, thereby ensuring a more robust and nuanced understanding of how cognitive skills, language barriers, and BEP interact to impact the academic performance of rural Latine youth. Following Creswell and Plano Clark (2017), integration was achieved through methodological triangulation at the interpretation stage. Quantitative results provided statistical patterns regarding predictors of academic achievement, while qualitative data explained the mechanisms and lived experiences underlying these patterns.

#### Quantitative analysis

Descriptive statistics, correlations, and multiple linear regression models were tested using SPSS 29. Initial checks for assumptions (linearity, independence, homoscedasticity, and normality of residuals) were conducted to ensure the analysis was robust. Six multiple linear regression models were specified to examine the unique contributions of CEF, BEP, and academic outcomes (GPA, Math, and ELA scores). In all models, demographic variables (ELL status, sex, and age) were included as covariates. Considering that parents and teachers reported on the youth's BEP, and to account for potential source differences, models were run separately by reporter. Analyses were conducted using complete case analysis for each dependent variable. The first three models separately assessed the three different academic outcomes using the parent report of BEP. Model 1 examined GPA T2, with predictors including inhibitory control, cognitive flexibility, receptive vocabulary, prosocial behavior, and behavioral and emotional problems at T1. Model 2 replicated Model 1 but used math T2 scores as the dependent variable. Model 3 replicated Model 1 but used ELA T2 scores as the dependent variable. Models 4–6 used teacher reports of BEP, but mirrored models 1–3 regarding academic outcomes (Model 4 assessed GPA, Model 5 assessed math scores, and Model 6 assessed ELA scores).

#### Qualitative analysis

Thematic analysis of semi‐structured interviews was conducted using NVivo 14, following the five‐phase process described by Braun and Clarke (2006): familiarization with data, initial coding, theme identification, review of themes, and definition/naming of themes. Interviews were transcribed verbatim and translated into English. Two independent bilingual coders (a bilingual undergraduate student and the first author) coded the data, after which they met to compare codes, discuss discrepancies, and establish interrater reliability exceeding Kappa = 0.95. To enhance analytic rigor, the research team engaged in reflective discussions that allowed for interrogation of interpretations and examination of potential biases (Lincoln & Guba, 1985). Inductive coding was employed to identify emergent patterns and themes directly from the participants' narratives without imposing a predefined structure. Subsequently, a deductive coding phase was conducted, informed by the study's central research questions and conceptual framework. This step enabled the classification and refinement of the initial codes into theoretically meaningful domains, ensuring alignment with the constructs explored in the study's qualitative and quantitative components (Saldaña, 2021). Finally, a process of reflexive adjustment was undertaken, wherein the inductive codes were revisited and reorganized in light of the deductive categories and analytic discussions. This iterative refinement led to the establishment of a hybrid code structure. While the deductive domains guided the development of the coding framework, the reporting of findings was ultimately organized around a central cross‐cutting pattern that more effectively captured the coherence of participants' experiences. Subthemes were then used to elaborate on the dimensions of this overarching pattern. This reporting strategy (Table 5) allowed for a more integrated and interpretive narrative, while still reflecting the foundational structure derived from the study's theoretical constructs. Thematic comparisons were also made between ELL and non‐ELL students, as well as across gender.

## RESULTS

### Quantitative results

Correlations among key study variables are shown in Table 1. The executive functioning tests (DCCS and FICA) were significantly correlated with Math and ELA scores, but only cognitive flexibility (DCCS) was significantly correlated with GPA. BEP was negatively associated with academic outcomes. ELL and MEP status were negatively correlated with both CEF and academic variables.

**TABLE 1:** Descriptive statistics and zero‐order correlations among main variables (*N* = 178).

Predictor	Mean	SD	1	2	3	4	5	6	7	8	9	10	11	12	13
1 GPA T2	2.75	0.95													
2 Math T2	6467.31	95.90	.42**												
3 ELA T2	5480.26	90.13	.45**	.80**											
4 DCCS T1	7.2	1.68	.13	.39**	.36**										
5 FICA T1	7.87	1.11	−.04	.35**	.36**	.44**									
6 PV T1	0.48	0.01	.04	.17*	.10	.15*	.13*								
7 SDQ PR T1	1.67	0.22	−.31**	−.21*	−.16	−.06	.07	−.03							
8 PRO PR T1	1.48	0.34	.14	.01	−.02	−.01	−.12	.09	−.54**						
9 SDQ TR T1	26.31	5.5	−.25**	−.27**	−.24**	−.14*	−.06	−.05	.30**	−.17*					
10 PRO TR T1	12.37	2.38	−.20**	−.15*	−.07	−.07	−.11	−.13*	.10	−.17*	.56**				
11 Gender T1	0.53	0.5	−.12	−.01	−.12	−.02	.15*	.05	.20*	−.28**	−.03	−.03			
12 Age T1	12.22	1.58	.02	.18*	.26**	.23**	.23**	−.02	.12	−.31**	−.02	.10	−.00		
13 ELL T1	0.65	0.48	−.17*	−.46**	−.51**	−.22**	−.18**	−.01	−.11	.24*	.06	.08	.15*	−.17**	
14 MEP T1	0.33	0.47	−.25**	−.23**	−.26**	−.09	−.02	−.01	.06	.15	.12*	.15*	.06	−.16**	.18**

*Note*: Time 2: ELA, English Language Arts; GPA, Grade Point Average. Time 1: DCCS, cognitive flexibility; FICA, inhibitory control; PV, receptive vocabulary; SDQ, behavioral and emotional problems; PRO, prosocial behavior; PR, parent report; TR, teacher report; T1, Time 1; T2, Time 2. ELL, English Language Learner (0 = non‐ELL, 1 = ELL); MEP, Migrant Education Program (non‐MEP, 1 = MEP); Gender (0 = girls, 1 = boys).

* *p* < .05.

** p < .001.

The first three regression models used the parent report of general difficulties and prosocial behavior. Model 1 was statistically significant, *F*(9, 163) = 3.64, *p* < .001, explaining 16.7% of the variance in GPA (*R* ^*2*^ = .17). Significant predictors included the DCCS score at T1 (*b* = 0.16, *p* = .04, *η* ^2^ₚ = .07), indicating that greater cognitive flexibility was associated with higher GPA at T2, and SDQ (*b* = −0.27, *p* < .001, *η* ^2^ₚ = .01) suggesting that higher behavioral and emotional problems predicted lower GPA at T2. Model 2 examined Math performance at T2. The model was statistically significant, *F*(9, 140) = 8.72, *p* < .001, explaining 36% of the variance in Math scores (*R* ^*2*^ = .36). Significant predictors included the DCCS score (*b* = 22.46, *p* = .001, *η* ^2^ₚ = .00), indicating that greater cognitive flexibility was associated with better Math performance at T2; behavioral (*b* = −15.07, *p* = .032, *η* ^2^ₚ = .03), suggesting that more socioemotional problems predicted lower Math performance at T2; and ELL status (*b* = −81.26, *p* < .001, *η* ^2^ₚ = .18), indicating that ELL status was associated with significantly lower Math performance at T2. For ELA scores (Model 3), the regression model was also significant, *F*(9, 139) = 9.73, *p* < .001, explaining 39% of the variance (*R* ^*2*^ = .39). Significant predictors included the DCCS score (*b* = 17.65, *p* = .007, *η* ^2^ₚ = .001), indicating that higher cognitive flexibility was associated with better ELA performance at T2, and ELL status (*b* = −78.00, *p* < .001, *η* ^2^ₚ = .02), again suggesting an association of ELL status with lower scores on academic achievement at T2. To view the complete models, refer to the left panel of Tables 2, 3, 4.

**TABLE 2:** Multiple regression results predicting GPA from executive function, behavioral measures, and demographic variables using parents and teacher reports.

Predictor	Estimate	S.E.	95% CI	*p*	*η* ^2^ₚ
Parent report (*N* = 173)					
Intercept	3.06	0.13	[2.8, 3.32]	<.001	0.39
Cognitive flexibility	**0.16**	0.08	[0.01, 0.32]	.04	0.01
Inhibitory control	**−0.16**	0.09	[−0.34, 0.01]	.07	0
Receptive vocabulary	0.02	0.07	[−0.12, 0.15]	.81	0
Behavioral and emotional problems	**−0.27**	0.07	[−0.41, −0.13]	<.001	0.07
Prosocial behavior	0.11	0.07	[−0.02, 0.24]	.11	0.01
Gender^a^	−0.21	0.14	[−0.49, 0.07]	.14	0.02
Age	−0.04	0.08	[−0.19, 0.11]	.61	0
English language learner^b^	−0.11	0.15	[−0.4, 0.19]	.48	0
Migrant education program^c^	−0.2	0.16	[−0.52, 0.11]	.21	0.01
Teacher report (*N* = 91)					
Intercept	2.95	0.2	[2.56, 3.36]	.00*	0.44
Cognitive flexibility	**0.22**	0.11	[0.02, 0.43]	.04	0
Inhibitory control	−0.08	0.13	[−0.33, 0.17]	.51	0.02
Receptive vocabulary	−0.06	0.1	[−0.26, 0.14]	.55	0
Behavioral and emotional problems	**−0.28**	0.12	[−0.51, −0.05]	.02*	0
Prosocial behavior	0.09	0.13	[−0.17, 0.34]	.5	0.08
Gender^a^	0.01	0.21	[−0.41, 0.42]	.96	0
Age	0.1	0.11	[−0.12, 0.31]	.37	0
English Language Learner^b^	−0.18	0.22	[−0.61, 0.25]	.41	0
Migrant Education Program^c^	0.02	0.2	[−0.39, 0.42]	.94	0

*Note*: The parent report model was significant, *F*(9, 163) = 3.64, *p* < .001, *R* ^2^ = 0.17, adjusted *R* ^2^ = .13. The teacher report model was significant: *F*(9, 82) = 2.28, *p* < .001, *R* ^2^ = .20, Adjusted *R* ^2^ = .11. Boldface indicates statistically significant predictors at *p* < .05.

Abbreviations: CI, confidence interval; Estimate, unstandardized regression coefficient*; η* ^2^ₚ, partial eta‐squared; S.E., standard error.

^a^ Gender (0 = girls, 1 = boys).

^b^ English Language Learner (0 = non‐ELL, 1 = ELL).

^c^ Migrant Education Program (0 = non‐MEP, 1 = MEP).

* *p* < .05.

**TABLE 3:** Multiple regression results predicting math scores from executive function, behavioral measures, and demographic variables using parents and teacher reports.

Predictor	Estimate	S.E.	95% CI	*p*	*η* ^2^ₚ
Parent report (*N* = 150)					
Intercept	6530.37	12.44	[6504.95, 6602.07]	<.001*	0.99
Cognitive flexibility	**22.46**	6.9	[8.82, 36.10]	.00*	0
Inhibitory control	1.73	7.99	[−14.06, 17.52]	.83	0.04
Receptive vocabulary	7.47	6.5	[−5.38, 20.33]	.25	0
Behavioral and emotional problems	**−15.07**	6.96	[−28.82, −1.31]	.03*	0.03
Prosocial behavior	1.76	6.43	[−10.96, 14.48]	.79	0
Gender^a^	11.57	13.08	[−37.43, 14.29]	.38	0.01
Age	−3.53	8.14	[−19.63, 12.57]	.67	0
English Language Learner^b^	**−81.26**	14.54	[−110.01, −52.52]	<.001*	0.18
Migrant Education Program^c^	−13.2	14.32	[−41.52, 15.12]	.36	0.01
Teacher report (*N* = 81)					
Intercept	6544.57	22.65	[6499.41, 6589.73]	<.001*	0.99
Cognitive flexibility	**22**	10.47	[1.11, 42.88]	.04*	0.02
Inhibitory control	9.5	12.56	[−15.55, 34.55]	.45	0.08
Receptive vocabulary	5.51	10.33	[−15.09, 26.12]	.59	0
Behavioral and emotional problems	−20.51	14.16	[−48.73, 7.72]	.15	0
Prosocial behavior	0.98	14.45	[−27.83, 29.79]	.95	0.03
Gender^a^	2.84	22.1	[−41.23, 46.91]	.9	0
Age	5	13.91	[−22.74, 32.74]	.72	0
English Language Learner^b^	**−91.13**	24.63	[−140.24, −42.01]	<.001*	0.17
Migrant Education Program^c^	−15.09	20.16	[−55.29, 25.11]	.46	0.02

*Note*: The parent report model was significant, *F*(9, 140) = 8.72, *p* < .001; *R* ^2^ = 0.36; *R*_adj_^2^ = 0.32. The teacher report model was significant: *F*(9, 71) = 4.22, *p* < .001; *R* ^2^ = 0.35; *R*_adj_^2^ = 0.27. Boldface indicates statistically significant predictors at *p* < .05.

Abbreviations: *η* ^2^ₚ, partial eta‐squared; CI, confidence interval; Estimate, unstandardized regression coefficient; S.E., standard error.

^a^ Gender (0 = girls, 1 = boys).

^b^ English Language Learner (0 = non‐ELL, 1 = ELL).

^c^ Migrant Education Program (0 = non‐MEP, 1 = MEP).

* *p* < .05.

**TABLE 4:** Multiple regression results predicting ELA scores from executive function, behavioral measures, and demographic variables using parents and teacher reports.

Predictor	Estimate	S.E.	95% CI	*p*	*η* ^2^ₚ
Parent report (*N* = 149)					
Intercept	5558.22	11.69	[5535.11, 5581.33]	<.001*	0.998
Cognitive flexibility	**17.65**	6.41	[4.96, 30.33]	.01**	0.001
Inhibitory control	6.1	7.44	[−8.6, 20.8]	.41	0.039
Receptive vocabulary	1.91	6.06	[−10.07, 13.9]	.75	0.003
Behavioral and emotional problems	−10.25	6.47	[−23.05, 2.55]	.12	0.018
Prosocial behavior	−7.09	5.97	[−18.89, 4.72]	.24	0.011
Gender^a^	−15.57	12.17	[−39.62, 8.49]	.2	0.01
Age	6	7.57	[−8.96, 20.97]	.43	0.003
English Language Learner^b^	**−78**	13.53	[−104.76, −51.25]	<.001*	0.203
Migrant Education Program^c^	−17.47	13.31	[−43.78, 8.84]	.19	0.016
Teacher report (*N* = 81)					
Intercept	5566.27	19.83	[5526.73, 5605.82]	<.001*	0.99
Cognitive flexibility	14.28	9.17	[−4.01, 32.57]	.12	0
Inhibitory control	20.15	11	[−1.78, 42.09]	.07+	0.03
Receptive vocabulary	1.57	9.05	[−16.48, 19.61]	.86	0
Behavioral and emotional problems	−18.89	12.4	[−43.61, 5.83]	.13	0
Prosocial behavior	−1.98	12.65	[−27.21, 23.25]	.88	0.03
Gender^a^	−36.13	19.36	[−74.72, 2.47]	.07+	0.03
Age	21.5	12.18	[−2.8, 45.79]	.08+	0.04
English Language Learner^b^	**−73.67**	21.57	[−116.68, −30.66]	.00*	0.17
Migrant Education Program^c^	−14.77	17.65	[−49.97, 20.43]	.41	0.01

*Note*: The parent report model was significant: *F*(9, 139) = 9.73, *p* < .001; *R* ^2^ = .39; *R*_adj_^2^ = .35. The teacher report model was significant: *F*(9, 71) = 5.65, *p* < .001; *R* ^2^ = 0.42; *R*_adj_^2^ = 0.34. Boldface indicates statistically significant predictors at *p* < .05.

Abbreviations: *η* ^2^ₚ, partial eta‐squared; CI, confidence interval; Estimate, unstandardized regression coefficient; S.E., standard error.

^a^ Gender (0 = girls, 1 = boys).

^b^ English Language Learner (0 = non‐ELL, 1 = ELL).

^c^ Migrant Education Program (0 = non‐MEP, 1 = MEP).

^+^ *p* < .10.

* *p* < .05.

^**^ *p* < .001.

Models 4–6 used the teacher's report of general difficulties and prosocial behavior. Model 4 examined GPA and yielded a statistically significant regression model, *F*(9, 82) = 2.28, *p* = .025, explaining 20% of the variance in GPA (*R* ^*2*^ = .20). Significant predictors were the DCCS score (*b* = .22, *p* = .04, *η* ^2^ₚ = .00), indicating that greater cognitive flexibility was associated with higher GPA at T2, and SDQ (*b* = −.28, *p* = .017, *η* ^2^ₚ = .00), suggesting that higher levels of behavioral and emotional problems predicted lower GPA at T2. Model 5 examined Math performance. The model was statistically significant, *F*(9, 71) = 4.22, *p* < .001, explaining 35% of the variance in Math scores (*R* ^*2*^ = .35). Significant predictors included the DCCS score (*b* = 22, *p* = .039, *η* ^2^ₚ = .02), indicating that greater cognitive flexibility was associated with better Math performance at T2, and ELL status (*b* = −91.13, *p* < .001, *η* ^2^ₚ = .17), suggesting that ELL status was associated with significantly lower Math performance at T2. Model 6 examined ELA scores. The model was statistically significant, *F*(9, 71) = 5.65, *p* < .001, explaining 42% of the variance in ELA scores (*R* ^*2*^ = .42). The only significant predictor was ELL status (*b* = −73.67, *p* = .001, *η* ^2^ₚ = .17), indicating that students classified as ELLs performed significantly lower in ELA at T2. To view the complete models, refer to the right panel of Tables 2, 3, 4.

### Qualitative findings

The qualitative narratives offer an account of how students navigate the classroom, expectations, academic engagement, and school‐based stressors, particularly under the pressures of English‐dominant instructional settings. Interviews were thematically analyzed in alignment with the study's focus on language proficiency, BEP, and CEF demands, comparing by ELL status and student gender. Organized around three interrelated themes, the qualitative analysis highlights students' firsthand accounts of comprehension breakdowns, emotional strain, and cognitive coping strategies that shaped their educational experiences. Specifically, three themes emerged from the interview data: (1) navigating comprehension barriers and participation avoidance; (2) managing emotional strain and stress responses; and (3) deploying cognitive coping strategies to sustain academic engagement despite linguistic and emotional challenges. These themes are summarized in Table 5.

**TABLE 5:** Themes, subthemes, and representative quotes (*n* = 47, ELL = 28, Non‐ELL = 19).

Theme	Definition	Representative quotes	Sample *n* (%)
Navigating comprehension barriers and participation avoidance			
Comprehension gaps and academic friction	Students encountered difficulty understanding academic content due to limited English proficiency	“The teacher talks, and I miss words. Then I miss what comes after. It's like I'm trying to build with missing pieces.” (C1028, ELL boy)	22 (47%)
Passive participation as a coping mechanism	Many students struggled with comprehension by avoiding participation, appearing disengaged, or remaining silent	“Sometimes I just look like I'm working so they don't ask me anything. I don't want to mess up in front of everyone.” (C1060, ELL girl)	18 (38%)
Emotional responses to language‐based strain in school life			
Emotional vulnerability in the classroom	Students described anxiety and shame in classroom language interactions, often leading to withdrawal	“It feels like my brain is always tired. Like I'm running but not getting anywhere.” (C1010, ELL boy)	20 (43%)
Lingering stress and emotional exhaustion	Students experienced cumulative fatigue and emotional burnout due to constant academic pressure	“It feels like my brain is always tired. Like I'm running but not getting anywhere.” (C1010, ELL boy)	17 (36%)
Regulating effort and coping with cognitive overload			
Task confusion, memory lapses, and systemic misalignment	Students reported issues with managing assignments due to unclear instructions and memory strain	“I had it on my computer but forgot to turn it in. Then it disappeared and I got an F.” (C1058, ELL boy)	19 (40%)
Stress responses and temporary withdrawal	Some students briefly disengaged to manage stress, then re‐engaged once they were calmer	“When I get too nervous, I just stop. Then later I try again when it's quiet.” (C1061, ELL girl)	15 (32%)
Help‐seeking, sibling scaffolding, and self‐rescue	Students used peer, sibling, or self‐made strategies to manage demands	“My older brother tells me to start with the easy one first. That helps me a lot.” (C1034, ELL boy)	21 (45%)

#### Navigating comprehension barriers and participation avoidance

Limited English proficiency was a consistent and central challenge for most participants classified as ELL (*n* = 22/28, 79%), shaping how they approached classroom activities, understood content, and decided whether to participate. Across interviews, ELL students described how gaps in comprehension affected not only their academic performance but also their confidence, autonomy, and sense of belonging. This theme revealed two interlinked patterns: (1) challenges in understanding teacher discourse and instructional content and (2) a subsequent tendency to withdraw from verbal participation and interaction.

##### Comprehension gaps and academic friction

Most of the 28 ELL students (*n* = 16/28, 57%) reported language difficulties that created a sense of unpredictability and loss of control during class instruction. The classroom was experienced not as a learning space, but as a source of persistent confusion. In contrast, non‐ELL peers (*n* = 11/19, 58%) described feeling confident in asking for clarification or engaging in discussion, showing how fluency enabled smoother academic continuity and classroom integration. When teachers used unfamiliar vocabulary or rapid speech, especially in subjects such as science or social studies, ELL students reported difficulty processing the information in real time. This not only disrupted learning but fostered emotional distress as demonstrated by one girl who stated: “I learn it at home, but then the teacher talks fast, and it is like my brain gets stuck. I feel nervous and just wait until it's over” (*ELL girl, 12 years, C1023*). Another participant shared a comparable experience: “It's not just hard to read the words. It's hard because you're thinking about how everyone is looking at you, waiting for you to mess up” *(ELL boy, 12 years, C1071)*.

##### Passive participation as a coping mechanism

Faced with frequent moments of misunderstanding, ELL students commonly adopted survival strategies rooted in silence, observation, or reliance on bilingual mediators. Several boys explained that they waited for cues from classmates rather than risk making errors in public: “The teacher uses big words. I just don't know what those mean yet. Sometimes [tests are] hard for me, because of the big words that they give us” (ELL boy, C1026). This strategy, waiting silently rather than asking, was reported by most ELL students (*n* = 18/28, 64%), with girls especially likely to describe emotional responses such as anxiety or embarrassment linked to language struggles (*n* = 12/15, 80%). This is illustrated by one girl who remarked: “Sometimes I tell them I don't know the answers, or I tell them I can't because I don't know much English. It's kind of embarrassing (ELL girl, C1022).”

In contrast to their ELL peers, non‐ELL students described classroom comprehension as largely unproblematic. For example, one non‐ELL boy (C1011) explained, “My teacher helps me when I don't get it the first time. She explains it again. I usually understand it after that.” This response reflects the overall trend among non‐ELL participants, 15 out of 19 (79%) of whom indicated that instructions were generally accessible and clarification, when needed, was readily obtained. A notable disparity emerged in how instruction was experienced across language groups, as non‐ELL students did not report recurring confusion or anxiety related to academic comprehension. Further, although ELL students often resorted to silence as a means of avoiding linguistic exposure, non‐ELL students rarely reported similar hesitation. One non‐ELL girl (C1049) shared, “If I don't know something, I ask my teacher or my friend. We figure it out together.” This sentiment was echoed by 17 of the 19 non‐ELL students (89%), most of whom expressed confidence in seeking clarification without fear of judgment. The discrepancy in comfort levels underscores how language proficiency intersects with participation dynamics, influencing not only who speaks but also who feels safe enough to do so.

These recurring moments of avoidance and emotional discomfort suggest more than just temporary misunderstandings. Instead, they illustrate a broader process of classroom disengagement. Language limitations contributed to the internalization of academic insecurity, altering how students perceived their intellectual capacities and shaping their long‐term self‐concept as learners. For many ELL students, classroom participation was not an opportunity for engagement but a risk of exposure. Students repeatedly faced linguistic confusion and its social consequences, so their classroom identities were increasingly characterized by invisibility or passivity. This pattern illustrates how language barriers extend beyond decoding content; they also mediate access to relationships, teacher validation, and educational opportunities. These emotional responses were not isolated but cumulative, developing over time and entangled with students' academic identity formation. As detailed in the following accounts, such reactions were described by a substantial portion of participant and reveal consistent gendered patterns in how emotional strain, was experienced and expressed.

#### Emotional responses to language‐based strain in school life

Language proficiency challenges extended beyond academic comprehension and shaped the emotional landscape of students' daily school experiences. While academic obstacles were more visible, students' narratives revealed persistent emotional consequences, ranging from anxiety and embarrassment to discouragement and fear of the future. Gender appeared to shape the tone and expression of these experiences. Girls tended to describe affective reactions more explicitly, using emotionally rich language such as “scared,” “tired,” or “ashamed.” While reporting comparable difficulties, boys often reframed their responses in pragmatic or subdued terms, describing disengagement as a matter of strategy rather than a sign of distress.

##### Emotional vulnerability in the classroom

Classroom interactions were emotionally charged for many ELL students (*n* = 18/28, 64%), especially for girls (*n* = 10/15, 67%). The fear of making mistakes, especially when speaking in English, often led to avoidance of verbal participation. Fear of exposure was particularly pronounced among girls, who expressed anxiety about comprehension as well as how peers perceived them. One girl explained: “When I had to read out loud, my heart started beating. I could feel everybody looking. I knew if I said one word wrong, they would all laugh. So, I looked at the paper and just stayed quiet” (ELL girl, C1043). Other students emphasized the social consequences of minor errors, such as one student who noted, “When I say something, and it's wrong, everyone looks at me weird. It makes me feel dumb, so I just stop talking and look at the floor” (ELL girl, C1057). Boys, although less expressive about emotional discomfort, reported similar forms of withdrawal. One boy said, “When I don't understand, I just wait for someone else to answer. I don't want to mess it up and have everyone look at me” (ELL boy, C1008).

In contrast, non‐ELL students rarely described verbal participation as emotionally risky. One girl (C1075, non‐ELL) remarked, “I don't get nervous to speak up. Even if I'm wrong, the teacher just helps me say it right.” Among non‐ELL students, 16 out of 19 (84%) described the classroom as a space where participation felt routine and emotionally safe. These accounts highlight a gap in how students experience the same classroom environment shaped mainly by language status.

##### Lingering stress and emotional exhaustion

In addition to momentary fear, many students described emotional fatigue as a persistent feature of school life. For ELL students, this fatigue accumulated from repeated misunderstandings, perceived failures, and the constant effort required to keep up. One girl said: “It's hard every day. I get tired of trying to understand everything. Sometimes I want to just stop because it never gets easier” (ELL girl, C1015). Girls were more likely to frame this fatigue as emotional exhaustion (*n* = 9/15, 60%), while boys emphasized disengagement (*n* = 7/13, 54%). One ELL boy (C1029) stated, “Sometimes it's annoying, but I just deal with it,” despite sharing his ongoing difficulties in following teacher instructions. The emotional toll was evident in both cases, whether articulated as sadness or framed through resignation. Some students expressed discouragement that their academic effort did not translate into recognition or success. One girl explained: “My stomach hurts when I know we have to read. I just want to go home” (ELL girl, C1067). Another boy captured a sense of helplessness: “I try and try, but when I still get things wrong, I just feel like maybe I'm not smart enough” (ELL boy, C1003).

Overall, 19 of the 28 ELL students (68%) reported experiencing emotional exhaustion or stress related to learning, with girls being more likely to link their language status to broader concerns about academic or future failure. One student offered a profoundly personal account: “Sometimes I get tired of learning, and I don't want to continue. However, I have to. I always tell myself, ‘I have to learn English.’ Nevertheless, I worry, what if I grow up and never teach it? That would be so hard” (ELL girl, C1051). Ten of the 15 ELL girls (67%) echoed this fear of linguistic stagnation, illustrating that language development was not just an academic goal, but also a source of personal anxiety and pressure. Non‐ELL students described their academic struggles in different ways. One non‐ELL boy (C1050) explained, “Sometimes it's hard, but I don't really stress about it. If I forget something, I just ask the teacher or finish it later.” Only 16% of non‐ELL participants referenced emotional strain, and none associated it with identity or self‐worth. Stress, when present, was described as temporary and linked to workload, not comprehension or public performance.

Language‐based emotional strain also intersected with students' social positioning. Several ELL students described being excluded, mocked, or bullied due to their accents or English proficiency. One ELL girl (C1018) recounted, “I was happy before, but that day was terrible. One boy was hitting me, [and] another was throwing pencils. I just cried and left. I didn't want to go back to class” (ELL girl, C1018).

These experiences reinforce the idea that emotional safety in school was not only shaped by pedagogy or academic support but also by peer dynamics and how students' language use affected their social standing. Emotional well‐being was contingent on having access to understanding teachers, bilingual peers, or culturally affirming relationships. Without these supports, language barriers became academic hurdles and emotional burdens that significantly shaped how students navigated their daily school experiences.

#### Regulating effort and coping with cognitive overload

As ELL Latine students navigated monolingual academic settings, their narratives reflected the mental strain of managing multiple cognitive demands, especially under linguistic friction, instructional pressure, and limited institutional scaffolding. Though they were not formally diagnosed with executive function difficulties, their interview accounts illuminated ongoing challenges in memory, organization, and emotional self‐regulation. These challenges did not signal apathy or inability; instead, they exposed the invisible labor ELL students performed daily to stay engaged.

##### Task confusion, memory lapses, and systemic misalignment

Many participants described experiences where they failed to submit homework, forgot assignments, or confused deadlines, not due to negligence, but rather because of cognitive overload and confusion with multistep tasks, particularly in digital formats. This difference highlights the strain on executive functioning that language processing demands in ELL youth. These scenarios, described by 19 of the 28 ELL students (68%), illustrate how instructional pacing and platform demands often exceeded what students could hold in working memory, mainly when simultaneously translating, interpreting instructions, and navigating new vocabulary. In school contexts where linguistic and cognitive demands are not fully recognized, these instances may be interpreted as carelessness or lack of effort, contributing to cycles of misunderstanding between students and instructional expectations.

One ELL student described: “I did the whole thing, like the slides and answers, but I didn't click submit. It was on my Chromebook, but it disappeared. Then I got an F, and I had to do it again. It made me feel like all my work didn't matter” (ELL boy, C1058). Similarly, a girl shared the tension between trying to keep up with fast‐paced subject transitions and falling behind: “They give us math, then reading, then science, and I try to remember everything. However, sometimes I just mix it up. Like I forget which one to turn in or which is due first” (ELL girl, C1031). Non‐ELL students described forgetfulness or task confusion, but these were typically framed as minor disruptions rather than persistent challenges. One non‐ELL girl (C1079) explained, “Sometimes I forget an assignment, but I just check Google Classroom. It's easy to find again.” Only 4 of the 19 non‐ELL students (21%) mentioned difficulty keeping track of assignments, compared to 21 of the 28 ELL students (75%) who reported repeated breakdowns in memory or sequencing.

##### Stress responses and temporary withdrawal

In the face of overwhelming demands, many students admitted to pausing or withdrawing, not as a form of avoidance, but as a self‐regulation mechanism to protect their focus and emotional stability. Of the 28 ELL students (50%), 14 (50%) described these forms of disengagement, with boys exhibiting this behavior more often. They described physical symptoms of stress, such as nervousness or headaches, often associated with multitasking, digital deadlines, or sudden classroom pressure. A boy described how falling behind in one subject led to an emotional and organizational cascade: “If I get behind in one thing, I kind of lose track of everything. Then I don't know where to start, and it just keeps going.” (ELL Boy, C1035). A girl also described this pressure. “When I get nervous because of too much homework, I take a break. I listen to music or go to my room and then come back. If I look at it all at once, I panic” (ELL girl, C1056). This girl's strategy, emotional reset before re‐engagement, demonstrates an adaptive understanding of her limits. However, their narratives emphasized that these were not permanent withdrawals but stress responses intended to regain cognitive clarity.

##### Help‐seeking, sibling scaffolding, and self‐rescue

Despite these challenges, students showed persistence and a strong capacity to identify and deploy support systems. Nearly all students (*n* = 24, 86%) described having someone (a sibling, teacher, or bilingual peer) who helped them break down instructions or manage assignments. For example, one girl described help from her older sister. “My sister helps me with the order. She says, ‘Do this one first, then this one.’ That helps me not get stressed. She explains the homework” (ELL girl, C1024). These micro‐strategies, asking for repetition, working alongside someone else, and organizing by task type, revealed student‐initiated forms of cognitive support. More than half of the ELL students (56%) discussed using such strategies, although their effectiveness depended on whether school environments allowed for time, patience, and relationships that were conducive to support. Even boys, who were more often reluctant to articulate emotional or cognitive overwhelm, noted the value of support: “My teacher helps when I ask. But sometimes I don't ask because I don't want to look like I don't know. So, I just try to guess. Or I wait for help later at home” (ELL boy, C1017).

These moments showed how reluctance to request assistance publicly often delays support, reinforcing the need for proactive scaffolding built into classroom routines. Non‐ELL students often described direct, unhesitating reliance on the teacher or peers when facing confusion. For example, one non‐ELL boy (C1053) noted, “I usually just ask the teacher or go to a friend. It is not a big deal. We always help each other out.” This attitude was typical among non‐ELL participants (16/19, 84%) who described help‐seeking as routine and emotionally neutral. By contrast, ELL students often described weighing social and emotional risks before seeking help, a pattern that points to the affective complexity of academic regulation.

While executive function challenges are presented as memory lapses or delayed submission, they were rooted in cognitive overload and stress. Students did not simply “forget” to complete tasks; they were managing multitiered mental demands while navigating language translation, teacher expectations, and shifting formats. Importantly, students rarely framed these difficulties as intrinsic flaws; instead, their accounts reflected a system that did not accommodate their processing pace or bilingual realities. The strategies students employed, self‐pacing, reliance on family, and help‐seeking, indicate that what may appear as behavioral issues or disengagement in classroom settings often masks sophisticated, albeit unrecognized, forms of regulation. These findings underscore the need to address how academic effort is assessed and how schools can effectively scaffold cognitive load for bilingual students. A visual synthesis of these mixed method patterns, including points of convergence and divergence between qualitative and quantitative findings, is presented in Table S1.

## DISCUSSION

We examined the influence of executive functioning, language proficiency, and behavioral‐emotional challenges on the academic performance of rural immigrant Latine youth in the Midwest. The findings provide partial support for the study hypotheses. As expected, executive functioning, particularly cognitive flexibility, emerged as a robust predictor of academic performance across GPA, Math, and ELA outcomes, supporting the hypothesis that executive skills play a central role in academic success among rural Latine youth. Behavioral and emotional problems were negatively associated with academic outcomes in several models, partially supporting the hypothesis that socioemotional challenges undermine academic performance. Finally, ELL status was strongly associated with lower standardized Math and ELA scores, but not with GPA, indicating a more nuanced pattern than originally hypothesized and underscoring the importance of distinguishing between standardized and classroom‐based indicators of academic functioning. Guided by the integrative risk and resilience model (Suárez‐Orozco et al., 2018) and PVEST (Spencer, 1999), we interpret these patterns as adaptation processes shaped by how students appraise school demands and mobilize coping responses over time.

A convergent mixed methods design enabled the identification of both statistical predictors and student‐expressed mechanisms influencing academic performance. While regression models clarified associations among executive functioning, behavioral adjustment, and academic metrics, student narratives added critical depth, revealing how daily language navigation, task regulation, and school participation are experienced. This integration offers a multidimensional view of students' learning conditions and highlights how classroom functioning unfolds in real world, linguistically demanding environments.

Notably, students' ELL status emerged as a powerful organizing force shaping their academic experience, cognitive effort, and emotional well‐being. Quantitative models revealed that ELL status was significantly associated with lower scores on standardized Math and English Language Arts assessments 1 year later, but not with GPA, indicating a discrepancy between formal test performance and the everyday academic effort reflected in teacher‐assigned grades (Callahan et al., 2010; Free et al., 2014). This finding was elaborated by students' qualitative accounts of navigating comprehension breakdowns, instructional overload, and digital platform confusion, challenges that often resulted in memory lapses and temporary withdrawal as a means of emotional regulation. Far from indicating apathy, these behaviors reflected the compounded executive burden of processing academic content while simultaneously translating language in real time (Roebers, 2017; Greenfader, 2019). Girls were more likely to articulate this strain in emotional terms like fear of failure, exhaustion, or hopelessness, while boys described disengagement in more instrumental terms. These narratives also highlighted how exclusionary peer interactions and linguistic stigma further intensified the academic and emotional load for ELL students (Slopen et al., 2023). From a PVEST perspective, these patterns indicate that behaviors often labeled as disengagement may instead represent coping responses to linguistic stress, shaped by repeated experiences of stigma and exclusion. Thus, ELL status operates as a multidimensional condition shaping students' cognitive, emotional, and social access to learning.

Across both qualitative and quantitative findings, gender‐based patterns also emerged in students' emotional and behavioral responses to language‐related academic stress. Latine girls, particularly those identified as English Language Learners (ELLs), more frequently expressed anxiety, embarrassment, and social apprehension in classroom settings, especially during verbal participation. By contrast, boys were more likely to adopt pragmatic withdrawal or silence without articulating emotional distress. These patterns align with existing literature, which indicates that girls tend to internalize school‐related stress, whereas boys may externalize or suppress such responses (Morrison et al., 2003; Taylor et al., 2019). Coping strategies such as passive participation and classroom silence were commonly used by girls as affective self‐protection in emotionally unsafe environments, strategies that may preserve self‐esteem in the short term but ultimately restrict academic engagement and linguistic growth (Carrero et al., 2017; Escamilla et al., 2014). These gender differences, therefore, reflect not only variations in affective processing but also distinct pathways of academic identity development and self‐regulation, underscoring the need for classroom environments that are both linguistically and emotionally safe and responsive to gendered patterns of vulnerability; in PVEST terms, these patterns represent distinct coping repertoires that develop in response to shared stressors and carry downstream implications for academic identity and engagement.

### Interpretive integration of quantitative and qualitative results

Convergences observed here suggest that achievement patterns reflect both structural constraints and students' active responses to them. Convergence was established by comparing regression models and qualitative themes side by side, identifying overlapping patterns in predictive strength and student‐reported mechanisms. A primary convergence between quantitative and qualitative data confirmed the strong influence of ELL status on academic performance. In the regression models, ELL status was a robust negative predictor of standardized Math and ELA outcomes but not GPA. This aligns with previous research indicating that language proficiency profoundly shapes students' ability to access curriculum content and perform on linguistically loaded assessments (Abedi, 2009; August & Shanahan, 2017; Snyder et al., 2017). Qualitative accounts contextualized these findings by documenting how limited English proficiency undermined classroom participation, comprehension, and students' confidence. Several students described being retained or demoted in grade placement due to limited English, whereas others emphasized their difficulty understanding exam instructions and vocabulary.

A second area of convergence lay in the central role of executive functioning, particularly cognitive flexibility, as a predictor of academic success. The cognitive flexibility consistently predicted GPA, Math, and ELA scores across multiple regression models, confirming its significance as a foundational cognitive skill that supports academic engagement, consistent with other studies (Cumming et al., 2024; Roebers, 2017; Taylor & Ruiz, 2019). Qualitative narratives supported these findings, with students demonstrating metacognitive awareness, emotional regulation, and planning behaviors that reflected key aspects of executive functioning. For instance, students described their help‐seeking behaviors, study routines, and future‐oriented motivation, particularly when these were scaffolded by family or teacher support.

There was also convergence regarding the impact of behavioral and emotional problems and academic performance. Quantitative results showed that higher BEP, as reported by both parents and teachers, was negatively associated with GPA and Math scores (Goodman, 2001; Horan et al., 2016; Shi et al., 2021). These findings were echoed in the qualitative data, where students reported stress, anxiety, and social withdrawal in response to language barriers and academic demands. Girls, in particular, internalized distress through self‐doubt and withdrawal, whereas boys more frequently externalized frustration through disengagement or disruptive behavior patterns aligned with gender differences in coping (Taylor et al., 2019). Consistent with the integrative model of immigrant‐origin youth (Suárez‐Orozco et al., 2018), these findings suggest that students' academic engagement must be interpreted within broader structures of opportunity and risk. From a PVEST perspective (Spencer, 1999), the coping strategies students described, such as silence, avoidance, or selective participation, reflect meaning‐laden responses to stress rather than fixed individual traits.

### Divergences between quantitative and qualitative findings

Divergences emerged through cross‐strand analysis, where findings from regressions were compared with students' first‐person narratives. Rather than inconsistencies, these reflect complementary insights that deepen the interpretation of student experience. Most notably, there was a discrepancy between standardized test scores and GPA. ELL status was a negative predictor of standardized scores but not GPA, which may reflect the broader scope of academic engagement embedded in GPA, such as effort, participation, and relational dynamics, that standardized tests often overlook. Additionally, GPA reflects performance across all academic subjects rather than only Math and English Language Arts and therefore captures a broader range of classroom competencies, including effort, participation, and relational dynamics. Because these everyday indicators of academic functioning are less dependent on momentary linguistic precision than standardized assessments, GPA may be less sensitive to the immediate effects of language‐based barriers. GPA reflects performance across all academic subjects, not only Math and English Language Arts, thereby capturing a wider set of competencies and classroom behaviors. This broader academic scope may further explain why its association with EF differed from the more domain‐specific standardized assessments. This divergence mirrors ongoing critiques that standardized assessments systematically disadvantage multilingual students by prioritizing linguistic accuracy over conceptual understanding or resilience (Callahan, 2013; Free et al., 2014). Moreover, qualitative data collected at Time 1 revealed that students perceived greater success in coursework and homework, particularly when bilingual support was available. While academic outcomes were assessed 1 year later (Time 2) and may reflect additional influences not directly measured in this study (e.g., changes in teachers, courses, or instructional contexts), these narratives help contextualize why GPA diverged from standardized test outcomes. As a cumulative classroom measure, the GPA may better capture sustained effort, relational trust, and teachers' responsiveness to student progress, dimensions that are largely absent from timed, linguistically loaded state assessments.

A second divergence involved the interpretation of BEP. Rather than indicating oppositionality or lack of engagement, students described these behaviors as strategic forms of self‐regulation in linguistically incongruent settings. While SDQ data linked behavioral difficulties to lower academic outcomes, interview data challenged the assumption that these behaviors reflect deficits in learning. Instead, qualitative findings illustrated how such behaviors were contextually adaptive responses to linguistic exclusion, academic stress, or fear of social ridicule (Carrero et al., 2017; Slopen et al., 2023). For example, students described staying quiet in class to avoid mockery or disengaging from homework when they could not understand the instruction—behaviors that might be pathologized in surveys, but represent survival strategies in unsupportive or linguistically inaccessible environments.

Another notable divergence exists around vocabulary proficiency and classroom engagement. While quantitative analyses showed no significant relationship with GPA and only modest links with math and ELA achievement, qualitative findings revealed a clear gap in vocabulary comprehension that acted as a daily barrier for ELL students. ELL participants described difficulties seeking help and navigating unfamiliar academic language, whereas non‐ELL peers routinely expressed confidence in asking for clarification and engaging with new vocabulary. The mismatch between students' test‐based vocabulary scores and their classroom experiences indicates that traditional assessments may fail to capture the nuanced, context‐driven nature of vocabulary use, especially for ELLs navigating both content and language acquisition. (Gándara & Contreras, 2009; Greenfader, 2017). Methodologically, the picture vocabulary test captures receptive vocabulary in a controlled setting. By contrast, classroom communication requires simultaneous listening, processing, and social interaction, considerably more challenging tasks for emergent bilinguals (Abedi, 2009). This finding is consistent with second language acquisition literature, which emphasizes that vocabulary growth among emergent bilinguals unfolds through situated discourse and ongoing negotiation of identity and meaning (Escamilla et al., 2014). Critically, this negotiation and active engagement process in classroom language tasks also depends on reducing the affective filter, a psychological barrier linked to anxiety, insecurity, and perceived incompetence that can inhibit participation (Krashen, 1982). These findings underscore the need for more contextual vocabulary measures and emotionally safe classroom environments where ELLs feel comfortable taking risks and coping with the demands of real‐world academic interaction.

Last, quantitative analysis revealed no significant relationship between prosocial behavior and academic outcomes, whereas qualitative data highlighted the importance of peer and teacher relationships in mediating academic success. Many students described how bilingual peers helped them understand assignments, or how patient, culturally responsive teachers supported their engagement (Ruiz et al., 2024; Suárez‐Orozco et al., 2018; Taylor et al., 2022). While difficult to quantify, these relational dynamics shaped students' motivation and ability to persist academically, underscoring the need for a broader conceptualization of the school environment's role in supporting ELL students. These divergences do not undermine the quantitative findings; instead, they reveal the limits of standardized measures in capturing the lived realities and adaptive capacities of students navigating complex educational environments. Such insights reflect the strength of convergent mixed methods in surfacing both measurable trends and the contextual nuances behind them.

### Critical synthesis and unique contributions of the study

This study contributes to the literature on rural Latine students by employing a convergent mixed methods design to provide a multidimensional account of academic performance. Addressing cognitive predictors and lived experience, this approach clarifies how executive functioning, language proficiency, and emotional regulation shape learning trajectories among youth in English‐only instructional contexts. A central convergence emerged around executive functioning. Quantitative models identified cognitive flexibility as a consistent predictor of GPA, as well as scores in Math and ELA. Students' accounts reinforced this pattern, describing metacognitive behaviors such as organizing tasks, monitoring effort, and adapting under pressure. These strategies were especially effective when reinforced by family or teacher support. This finding is consistent with second language acquisition literature, which emphasizes that vocabulary growth among emergent bilinguals unfolds through situated discourse and ongoing negotiation of identity and meaning, supporting the claim that executive functioning makes a meaningful contribution.

At the same time, several divergences deepened the interpretation. While ELL status predicted lower standardized test scores, it did not significantly affect GPA, suggesting that GPA captures additional dimensions of engagement, including effort and relational rapport. Similarly, behavioral difficulties were negatively linked to academic performance in the survey data. However, interviews revealed that behaviors like silence or withdrawal were often contextually adaptive and used to manage linguistic stress rather than defiance or disengagement. Another divergence appeared in the role of prosocial behavior. While youth‐reported prosocial tendencies were not significant in regression models, students frequently described receiving peer assistance, sibling help, and teacher guidance as crucial to comprehension and motivation. This distinction suggests that while the prosocial behaviors of focal students may not directly predict their academic outcomes, the support they receive from others plays a critical role in their learning process. These supports were described by over two‐thirds of participants and appear to mediate learning in ways not easily captured through traditional instruments. Finally, the study highlights how bilingualism is experienced not only as a skill but also as a cognitive and emotional challenge. Students' reflections on translating, misunderstanding, or feeling delayed offer important context for interpreting scores on linguistically loaded assessments. By centering students' school experiences, the study broadens understanding of how academic performance unfolds in real‐world conditions. Taken together, these findings advance an understanding of Latine ELL students' academic development that is consistent with the integrative model of immigrant‐origin youth (Suárez‐Orozco et al., 2018), foregrounding the structural and relational conditions shaping opportunity for learning. At the same time, PVEST provides a developmental lens to interpret students' narratives as meaning‐making processes in which coping responses emerge as contextually grounded strategies rather than individual deficits.

## LIMITATIONS AND FUTURE DIRECTIONS

This study offers meaningful insights into the academic experiences of rural Latine students, particularly ELL students, yet several limitations must be noted. First, although the convergent mixed methods design allowed for the integration of quantitative and qualitative findings, the two strands were implemented independently. As a result, integration occurred primarily at the interpretive stage, limiting participant overlap and opportunities for real‐time triangulation. Second, variation in response rates across informants introduced inconsistencies in the quantitative analyses. Specifically, fewer teachers than parents completed the behavioral assessment (SDQ), which may have reduced the robustness of teacher‐reported predictors in some regression models. Third, although predictor variables were assessed at Time 1 and academic outcomes were obtained 1 year later, the study does not constitute a fully longitudinal design capable of modeling causal or developmental change. While the temporal lag strengthens directional inference, unmeasured influences between waves—such as changes in teachers, classrooms, or instructional contexts—may have shaped academic outcomes.

Future research should address these limitations by employing longitudinal mixed‐methods designs with repeated measurements across multiple time points and stronger integration between qualitative and quantitative components. Such approaches would allow for examination of how executive functioning, emotional regulation, and academic engagement evolve over time, particularly during school transitions. Additionally, incorporating classroom observations or teacher interviews could enhance interpretation of behavioral patterns that students describe as adaptive but are often misclassified in survey‐based measures.

These findings offer several implications for theory, practice, and policy. Theoretically, the results support and extend integrative risk and resilience models by demonstrating how linguistic exclusion, emotional strain, and coping behaviors interact to shape academic outcomes. By incorporating PVEST, our findings underscore the profound connection between students' identity development and meaning‐making, as well as their experiences of academic risk, emotional vulnerability, and relational support within school contexts. Practically, the findings suggest that educational environments serving rural Latine youth must be intentionally structured to reduce cognitive overload and emotional stress associated with language demands. This includes pacing instruction, offering multimodal scaffolds, and building relational trust. Teacher preparation programs and school leadership initiatives should prioritize training in culturally and linguistically responsive pedagogy as well recognize gendered and language‐mediated expressions of academic distress. At the policy level, the gap between GPA and standardized assessments among ELLs suggests a misalignment between how student learning is experienced and how it is evaluated. This calls for greater investment in formative, multilingual assessments, and more equitable accountability systems that reflect the full scope of student growth and effort. In sum, a developmental and ecological lens reveals that academic success is shaped not only by language proficiency but also by the quality of school relationships, affective safety, and the degree to which schools affirm students' complex identities and strengths.

Supplementary materials are available in the Supplementary Section. Table S1 provides a visual synthesis of the points of convergence and divergence between the quantitative and qualitative findings presented in this study.

## AUTHOR CONTRIBUTIONS

All listed authors should have contributed to the manuscript substantially and have agreed to the final submitted version. Alejandro Baquero‐Sierra, Zoe E. Taylor, and Yumary Ruiz contributed to the conceptualization of the study. The methodology was developed collaboratively by Alejandro Baquero‐Sierra, Zoe E. Taylor, Alexia Carrizales, and Yumary Ruiz. Data curation and formal analysis were carried out by Alejandro Baquero‐Sierra, Zoe E. Taylor, and Yumary Ruiz, with additional support from Alexia Carrizales for the analysis. The investigation was conducted by all four authors. Zoe E. Taylor and Yumary Ruiz were responsible for validation, supervision, project administration, and securing funding. Alejandro Baquero‐Sierra led the writing of the original draft, whereas Zoe E. Taylor, Alexia Carrizales, and Yumary Ruiz contributed to reviewing and editing the manuscript.

## FUNDING INFORMATION

National Institutes of Minority Health Disparities (Grant no. R01MD014187).

## CONFLICT OF INTEREST STATEMENT

The authors declare that there are no actual or perceived conflicts of interest in the conduct, analysis, or reporting of this research.

## ETHICS STATEMENT

This study was approved by Purdue's Institutional Review Board #2019‐590.

## PATIENT CONSENT STATEMENT

All participants in this study were minors; therefore, informed consent was obtained from a parent or legal guardian prior to data collection, in accordance with institutional and ethical guidelines. Assent was also obtained from the participating children when developmentally appropriate.

## Supporting information


**Table S1.** Integration of quantitative and qualitative findings.

## Data Availability

The data that support the findings of this study are available on request from the corresponding author. The data are not publicly available due to privacy or ethical restrictions.
