# Supplementary material for: Language proficiency and academic achievement in rural and agricultural Latine youth: A mixed methods approach
Source: J Res Adolesc. 2026 Mar 23;36(1):e70166. doi: 10.1111/jora.70166 (PMC13006936; doi:10.1111/jora.70166)
Supplement: Supplementary file 1 — Table S1. Integration of quantitative and qualitative findings. [file JORA-36-0-s001.docx]

Supplemental Material.

Table S1.

Integration of Quantitative and Qualitative Findings.

| Topic | Quantitative Finding | Qualitative Finding | Convergence / Divergence | Implications |
| --- | --- | --- | --- | --- |
| GPA Performance | ELL students had lower GPA than non-ELL peers. | Students reported struggling to keep up academically due to language challenges and mental fatigue. | Convergence | Academic performance gaps are linked to cognitive overload and language strain, underscoring need for linguistic scaffolding in instruction. |
| Math Achievement | ELL status predicted significantly lower Math scores. | Math was described as more accessible when teachers used visual aids or peer explanations. | Partial Convergence | Language access, not just content, mediates performance. Instructional strategies should address linguistic barriers even in non-language subjects. |
| ELA Scores | ELL status strongly associated with lower ELA performance. | Students expressed fear, anxiety, and embarrassment when reading aloud or writing in English. | Convergence | Emotional responses to linguistic performance influence engagement; support should address both language and affective domains. |
| Participation in Class | Gender differences: girls participated less verbally than boys. | Girls reported fear of judgment, while boys described more disengaged or “silent” coping. | Convergence | Emotional safety affects participation differently by gender; SEL supports, and teacher responsiveness need to be gender-sensitive. |
| Executive Functioning (Organization/Focus) | Lower scores on working memory and organization among ELL students. | Students described difficulty remembering instructions, switching tasks, or managing time. | Convergence | Language demands impact executive functioning; instructional pacing and chunking can support ELL cognitive load. |
| Mental Health & Emotional Strain | Higher internalizing symptoms (e.g., anxiety) reported among ELL girls. | Girls expressed exhaustion, low motivation, and fear of failure; boys tended to minimize emotional impact. | Convergence (gender-patterned) | Mental health support must attend to gendered expressions of distress, especially under language-related academic pressure. |
| Teacher Relationships | Positive relationships predicted better academic outcomes across the sample. | Students emphasized how patient, bilingual, or supportive teachers helped them feel confident. | Convergence | Relationships serve as academic and emotional buffers; culturally and linguistically responsive teaching strengthens student outcomes. |
| Engagement vs. Vocabulary Skill | Vocabulary scores did not predict observed classroom engagement. | Students with low vocabulary still showed interest and effort; others with high vocabulary were disengaged. | Divergence | Engagement is not reducible to language proficiency; multiple factors (teacher, peers, self-belief) mediate motivation and behavior. |
